# Development and Assessment of Screening Nomogram for Biliary Atresia Based on Hepatobiliary Ultrasonographic Features

**DOI:** 10.3389/fped.2021.625451

**Published:** 2021-05-17

**Authors:** Shu Yang Dai, Yu Qi Sun, Ying Wu, Gong Chen, Song Sun, Rui Dong, Shan Zheng

**Affiliations:** ^1^Shanghai Key Laboratory of Birth Defect, Department of Pediatric Surgery, Children's Hospital of Fudan University, Shanghai, China; ^2^Key Laboratory on Public Health, Safety of the Ministry of Education, Department of Biostatistics, School of Public Health, Fudan University, Shanghai, China

**Keywords:** biliary atresia, nomogram, gamma-glutamyl transferase, ultrasound hepatobiliary abnormality, early screening

## Abstract

**Objectives:** Biliary atresia (BA) is a rare neonatal liver disease of which the early diagnosis remains a challenge for clinicians. Our center has established a nomogram diagnostic model based on clinical characteristics and liver function characteristics. We aim to develop and validate a nomogram that includes additional ultrasound and finds hepatobiliary abnormality with better BA early screening performance.

**Methods:** In this single-center, retrospective cohort analysis, 1,001 neonatal obstructive jaundice (NOJ) patients between 2012 and 2015 were enrolled. Multivariable analysis was used to identify clinical characteristics, laboratory liver function characteristics, and ultrasonic features that may early screen BA. A nomogram was developed to predict the probability of BA using multiple logistic regression analysis. This nomogram was subsequently validated using another cohort of 501 NOJ patients between 2015 and 2017. Calibration curve analysis and decision curve analyses were performed to evaluate and interpret the nomogram's clinical benefits.

**Results:** Gender, direct bilirubin (DB), alkaline phosphatase (ALP), gamma-glutamyl transpeptidase (GGT), fasting gallbladder visibility, fasting gallbladder filling, and common bile duct visibility were found to have profound statistical significance between the BA and non-BA groups (*P* < 0.05). The significant features were used to build the nomogram. The area under the receiver operating characteristic (ROC) curve (AUC) value of the novel nomogram (0.87) was superior to those of the former nomogram (0.83) and GGT alone (0.81) in the prediction of BA. The calibration curve revealed a close resemblance between the predicted and actual BA probabilities. Also, the net benefit from the decision curve analysis (DCA) of the nomogram (0.54) was superior to those of the former nomogram (0.49) and GGT alone (0.45) at 80% of threshold possibility.

**Conclusions:** The nomogram has demonstrated better performance for BA screening by including additional information of the US finding, holding a promising future as a non-invasive method for BA patients.

## Introduction

Biliary atresia (BA) is a devastating neonatal bile duct disorder in which continuous progressive fibrosis of the biliary tree in an infant would result in irreversible liver cirrhosis (1, 2). Currently, Kasai portoenterostomy is the only treatment besides liver transplantation, and most infant patients could regain bile drainage through this procedure (3). Without accurate early diagnosis and timely performance of Kasai portoenterostomy, progressive liver cirrhosis will cause infant death at the age of 2–3 years (4, 5). Therefore, the greatest challenge is screening and diagnosing the BA as early as possible in the early phase of life. Even today, the confirmative BA diagnosis still relies on invasive surgical cholangiography and intraoperative liver biopsy, which are relatively time-consuming and costly (6, 7).

Ultrasonography is a screening and diagnostic strategy for infantile cholestasis evaluation to exclude biliary atresia (8). Several specific sonographic features, such as the triangular cord sign (TCS), the gallbladder (GB) length, abnormal GB morphologic characteristics, the enlarged hepatic artery diameter, the presence of hepatic subcapsular flow, and the non-visualization of the common bile duct (CBD) have been acknowledged as valuable clues for biliary atresia diagnosis (9–13). Many studies have reported excellent specificity of these signs, despite insufficient sensitivity that varies widely among studies (14). From our experience, we believed that hepatobiliary abnormality found with ultrasound could facilitate early BA diagnosis to a certain extent, especially when a patient displayed with a high GGT level, and hepatobiliary ultrasound results show the visibility of GB and well GB filling (Supplementary Figure 1). In this case, US results implicated that the patient may have normal bile drainage, which means jaundice may not be caused by BA but instead by other causes of neonatal cholestasis.

Hence, to seek a method for early BA screening with higher accuracy, that is non-invasive, or that is less traumatic is in great demand. To our knowledge, no early screening model that combined the laboratory liver function markers and the US features together for early screening of BA has been reported to date. Our study aims to establish a new nomogram with additional US features and provide a novel and better model for early initial BA screening.

## Materials and Methods

### Data Acquisition

A total of 1,953 obstructive jaundice infant patients, who were suspected of BA, were admitted to the Children's Hospital of Fudan University's surgical department and underwent surgical cholangiography for screening from January 1, 2012 to November 30, 2015. Consequently, 1,001 out of 1,953 patients based on our inclusion and exclusion criteria were enrolled in this study for further analysis as the training data set. We enrolled 501 additional patients suspected of BA who underwent surgical cholangiography from December 1, 2015 to November 30, 2017, for the nomogram's independent external validation.

All the patients received routine hepatobiliary ultrasound examination (ACUSON SEQUOIA 512) before the surgery. Before the examination, patients fasted for 4–6 h and received sedation (oral chloral hydrate, 50 mg per kg) 30 min before administering the ultrasound examination. The detailed examination was carried out by two experienced pediatric radiologists with 5 and 10 years of experience in hepatobiliary ultrasound imaging, who reviewed and interpreted all of the infant patients' image features. The radiologists were blinded to the medical history of the patients. When the discrepancy was met, the final decision was made in consensus.

The inclusion criteria for the creation of the current nomogram were as follows: (1) infant patients who were confirmed of BA diagnosis through intraoperative cholangiography and displayed pathological features when intraoperative liver biopsies were performed; (2) not presenting with BA splenic malformation syndrome, bile duct dysplasia, or any other severe systematic deformity; and (3) patients underwent detailed hepatobiliary US examination with clear reports. Exclusion benchmarks were (1) patients with missing data and (2) patients with low US image quality and vague US reports.

**Figure 1:**
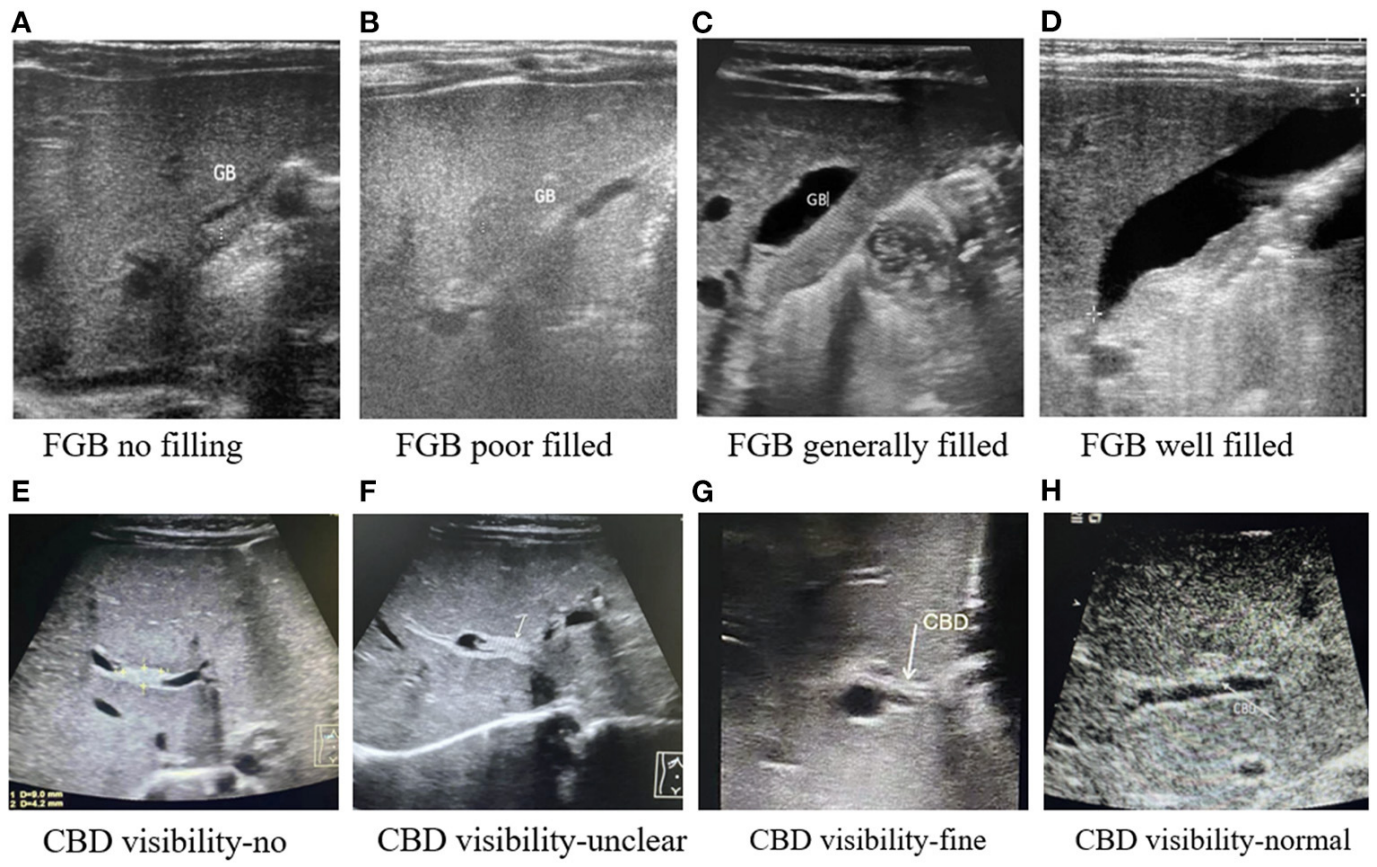
The classification of fasting gallbladder filing status and common bile duct visibility in ultrasonography from our center. **(A)** FGB no filling. **(B)** FGB poor filled. **(C)** FGB generally filled. **(D)** FGB well-filled. **(E)** CBD visibility-no. **(F)** CBD visibility-unclear. **(G)** CBD visibility-fine. **(H)** CBD visibility-normal.

Patient characteristics that were statistically relevant reported by our previous study, including gender, weight, alkaline phosphatase (ALP), aspartate transaminase (AST), total bilirubin (TBIL), direct bilirubin (DBIL), and gamma-glutamyl transpeptidase (GGT), were enrolled. Other newly added variables like albumin, total bile acid (TBA), and abnormal ultrasound hepatobiliary features like the size of the liver, fasting gallbladder visibility (FGBV), fasting gallbladder filling status (FGBF), and common bile duct visibility (CBDV) were enrolled for analysis in our study. The US hepatobiliary abnormality feature results were classified as follows: (1) size of the liver was divided into normal or enlarged; (2) fasting gallbladder visibility was divided into yes or no; (3) fasting gallbladder filling status was divided into no, poorly, generally filled, or well-filled; and (4) common bile duct visibility (CBD) was divided into invisible, unclear, fine, or normal. The classification of fasting gallbladder filing status and common bile duct visibility based on our center experience is shown in Figure 1.

The ethics committee of the Children's Hospital of Fudan University has approved this study. Informed consent was also obtained from the legal guardians of all the patients before enrollment in the study.

### Statistical Analysis

Statistical analysis was performed with commercially available R software version 3.6.3 (R Foundation for Statistical Computing). A *P*-value of <0.05 was considered significant; all tests were two-sided. Additionally, Fisher's exact test and Wilcoxon rank sum were used to analyze the categorical and continuous variables, respectively. The significant factors estimated by the analyses, as mentioned above, were further confirmed using univariate logistic regression. The variables that were found statistically significant in the univariate logistic regression analyses were used to construct the multivariate logistic regression. Moreover, the screening nomogram was obtained from the multivariate logistic regression. Stepwise regression analysis was performed to compare the Akaike information criterion (AIC) to select the best nomogram. Additionally, the depiction of the nomogram was performed by using the rms (version: 6.2-0, Developer: Frank E. Harrell Jr., Country: USA) package.

The logistic regression-based formula, which originated from the training cohort, was applied to the external validation cohort. The sensitivity and specificity determined by the Youden index were reported for comparison. The ROC curves were constructed by computing the sensitivity and specificity of increasing numbers of predicted probability in the prediction model. Then the AUC can be obtained by calculating the area under the ROC curve. Delong's test was used to compare the ROC curves. The screening performance of the nomogram in our paper was evaluated by the area under the receiver operating characteristic curve (AUC) and the calibration curve with 1,000 bootstrap samples. Besides, decision curve analysis was performed to assess the clinical benefits.

## Results

### Clinicopathologic Characteristics

The baseline clinicopathologic data showed a similarity between the training data set and the validation data set. The detailed data of clinicopathologic characteristics, such as age, body weight, ALB, ALP, AST, DBIL, GGT, GLB, TBA, TBIL, liver size, fasting gallbladder visibility, fasting gallbladder filling, and common bile duct visibility, are listed in Supplementary Table 1. In this data set, we identified that age and weight were not statistically significant, while gender, DBIL, ALP, GGT, and four indicators from the US were found to have a specific trend toward significance between the BA and non-BA groups (*P* < 0.05). Moreover, TBIL and AST levels were short of statistical significance between them (*P* > 0.05).

### Univariate Logistic Regression Analysis of Variables Significantly Associated With BA

As can be seen from Table 1, gender, log (ALP) (OR = 0.57, 95% CI = 0.38–0.81, *P* < 0.05), log (DBIL) (OR = 2.01, 95% CI = 1.33–3.12, *P* < 0.05), log (GGT) (OR = 3.37, 95% CI = 2.71–4.24, *P* < 0.05), fasting gallbladder visibility (yes: OR = 0.19, 95% CI = 0.09–0.34, *P* < 0.05), and fasting gallbladder filling (well-filled: OR = 0.14, 95% CI = 0.08–0.24, *P* < 0.05, generally filled: OR = 0.44, 95% CI = 0.28–0.70, *P* < 0.05) were identified to be statistically significant (*P* < 0.05).

**Table 1:** Univariate logistic regression analysis of the training data set.

		**OR**	**95% CI**	***P*-value**
Age		0.79	0.47–1.42	0.41
Gender	Female	Ref		
	Male	0.38	0.26–0.56	<0.0001
Size of liver	Normal	Ref		
	Enlargement	1.57	1.09–2.24	0.013
Fasting gallbladder visibility	No	Ref		
	Yes	0.19	0.09–0.34	<0.0001
Fasting gallbladder filling	No	Ref		
	Poor	0.78	0.49–1.26	0.30
	Generally filled	0.44	0.28–0.70	<0.00048
	Well-filled	0.14	0.08–0.24	<0.0001
Common bile duct visibility	Invisible	Ref		
	Unclear	3.17	1.11–13.35	0.060
	Fine	0.63	0.42–0.94	0.026
	Normal	0.27	0.16–0.47	<0.0001
log(weight)		1.02	0.51–2.04	0.96
log(ALB)		1.26	0.82–2.07	0.33
log(ALP)		0.57	0.38–0.81	0.0029
log(AST)		0.89	0.70–1.11	0.33
log(DBIL)		2.01	1.33–3.12	0.0012
log(GGT)		3.37	2.71–4.24	<0.0001
log(GLB)		1.24	0.62–2.48	0.54
log(TBA)		1.32	0.91–1.89	0.13
log(TBIL)		2.15	1.27–3.69	0.0045

*OR, odds ratio; Ci, confidential interval; Ref, reference*.

### Multivariate-Logistic Regression-Based Nomogram for BA Prediction

From the results of multivariate logistic regression analysis, gender (male: OR = 0.37, 95% CI = 0.23–0.58, *P* < 0.05), size of the liver (Enlargement: OR = 1.32, 95% CI = 0.84–2.06, *P* = 0.22), fasting gallbladder visibility (yes: OR = 0.27, 95% CI = 0.12–0.59, *P* < 0.05), fasting gallbladder filling (well-filled: OR = 0.21, 95% CI = 0.10–0.43, *P* < 0.05), common bile duct visibility (normal: OR = 0.30, 95% CI = 0.15–0.59, *P* < 0.05, Fine: OR = 0.46, 95% CI = 0.27–0.78, *P* < 0.05), log (ALP) (OR = 0.45, 95% CI = 0.27–0.72, *P* < 0.05), log (DBIL) (OR = 2.07, 95% CI = 0.85–3.63, *P* < 0.05), log(GGT) (OR = 3.11, 95% CI = 2.52–4.08, *P* < 0.05), and log(TBIL) (OR = 1.21, 95% CI = 0.51–3.02, *P* = 0.65) were associated with BA (Table 2). Because our goal is to build the prediction tool, the model was further refined by stepwise regression analysis based on AIC to select the final prediction model. The size of the liver and Log(TBIL) were removed. The final formula used to build nomogram is:

(1) $$\begin{array}{l} \text{logit}\left[\text{P}\left(\text{BA}\right)\right]=-0.811-0.993^{*}I\left(\text{gender }=\text{ male}\right)\\ +0.211^{*}I\left(\text{FGBF }=\text{ poor}\right)-0.311^{*}I\left(\text{FGBF }=\text{ generally filled}\right)\\ -1.511^{*}I\left(\text{FGBF }=\text{ well filled}\right)-1.330^{*}I\left(\text{FGBV }=\text{ yes}\right)\\ +0.947^{*}I\left(\text{CBDV }=\text{ unclear}\right)-0.730^{*}I\left(\text{CBDV }=\text{ fine}\right)\\ -0.120^{*}I\left(\text{CBDV }=\text{ normal}\right)-0.804^{*}\log\left(\text{ALP}\right)\\ +0.734^{*}\log\left(\text{DBIL}\right)+1.149^{*}\log\left(\text{GGT}\right) \end{array}$$

**Table 2:** Multivariate logistic regression analysis of factors associated with BA in the training data set.

		**OR**	**95% CI**	***P*-value**
Gender	Female	Ref		
	Male	0.30	0.23–0.56	<0.0001
Size of liver	Enlargement	1.32	0.84–2.06	0.22
Fasting gallbladder filling	No	Ref		
	Poor	1.32	0.84–2.06	0.55
	Generally filled	1.20	0.65–1.26	0.24
	Well-filled	0.21	0.10–0.43	<0.0001
Fasting gallbladder visibility	No	Ref		
	Yes	0.27	0.12–0.59	0.0014
Common bile duct visibility	Invisible	Ref		
	Unclear	2.42	0.75–10.98	0.18
	Fine	0.46	0.27–0.78	0.0046
	Normal	0.30	0.15–0.59	0.00056
log(ALP)		0.45	0.27–0.72	0.0016
log(DBIL)		2.07	0.85–3.63	0.087
log(GGT)		3.11	2.52–4.08	<0.0001
log(TBIL)		1.21	0.51–3.02	0.65

*OR, odds ratio; Ci, confidential interval; Ref, reference*.

### Establishment of the Nomogram and External Validation

**Figure 2:**
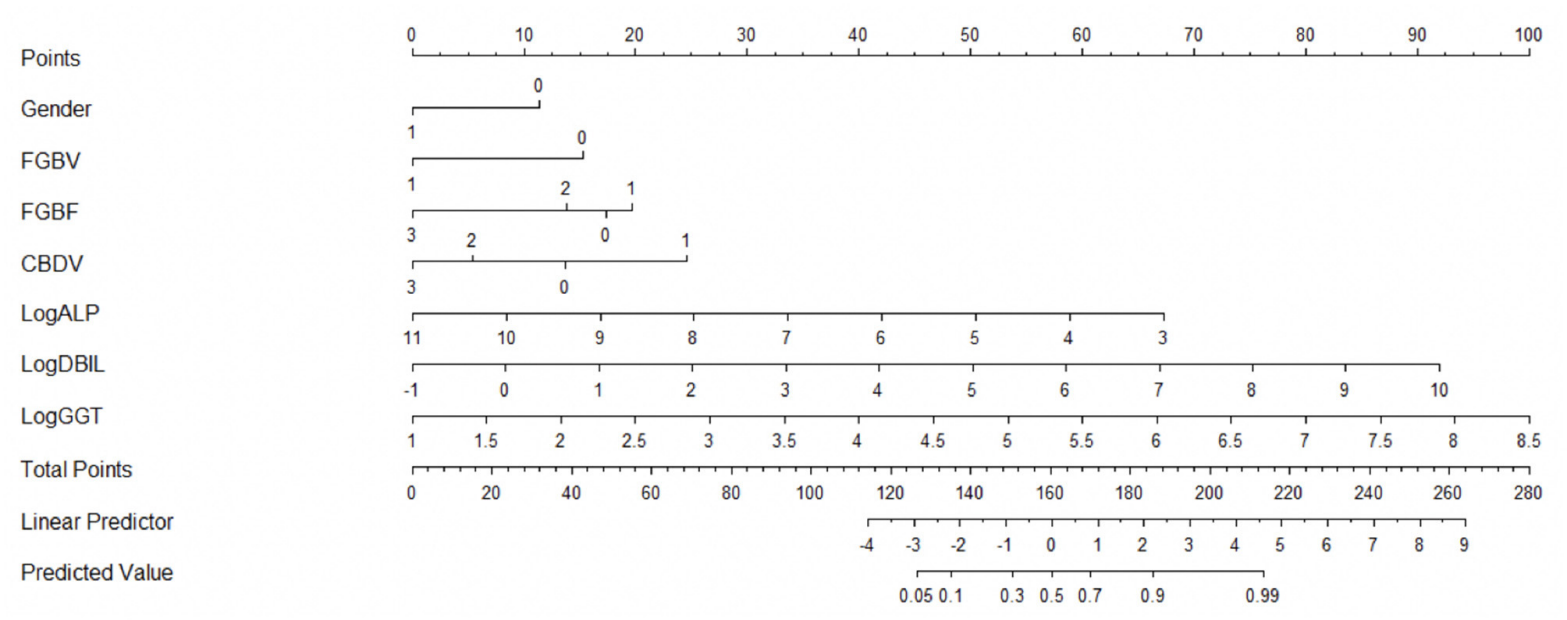
Novel nomogram [(Gender: 0 = Female, 1 = Male; FGBV (Fasting gallbladder visibility): 0 = No, 1 = Yes; FGBF (Fasting gallbladder filling): 0 = No, 1 = Poor, 2 = Generally Filled, 3 = Well-filled; CBDV (Common bile duct visibility): 0 = No, 1 = Unclear, 2 = Fine, 3 = Normal)].

**Figure 3:**
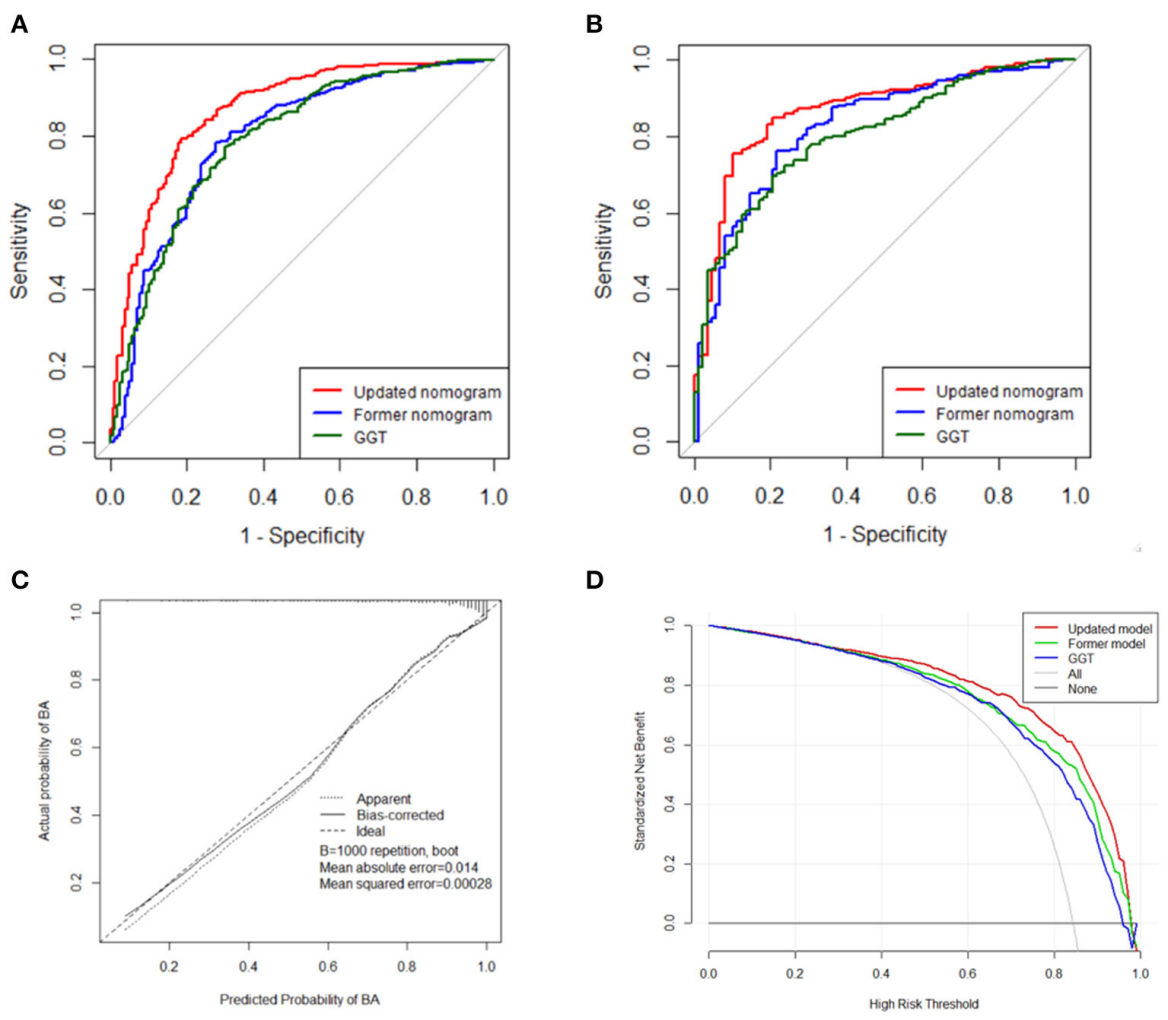
The receiver operating characteristic (ROC) curve of the novel nomogram and comparison with former nomogram and single GGT predictor in training data set **(A)** and validation data set **(B)**. Novel nomogram-predicted probability of BA in validation data set **(C)**. Decision curve analysis of the three predictive models for BA in validation data set **(D)**.

The novel nomogram is depicted in Figure 2. There were a total 11 of rows in this nomogram, and the included variables were represented from rows 2–8. Based on an individual patient's clinicopathologic characteristics, each of the seven predicting variables is allocated to a numerical value from the corresponding row. Each point from the seven variables was added for the final scores, which were showed in row 9 and revealed BA risk possibility. Thus, the nomogram could calculate the predicted BA probability for an individual patient. For a patient who was suspected of BA, if the gender of the patient is male, then we draw a vertical line upwards where the gender of the nomogram is 1, and the corresponding score is 0. If the same patient's FGBV indicates yes, the corresponding score is 0. Then, we repeat this step to find the corresponding score of each variable. Finally, by adding up the scores of all seven variables to get the patient's total score and then drawing a vertical line down, we can know the predicted value of this patient. The nomogram demonstrated good accuracy in the early screening of BA in the training data set, with an AUC of 0.86 (95% CI = 0.83–0.90, Sensitivity = 0.82, Specificity = 0.79), which was higher than those of the former nomogram (AUC = 0.79, 95% CI = 0.75–0.84, Sensitivity = 0.73, Specificity = 0.78) and GGT alone (AUC = 0.79, 95% CI = 0.75–0.84, Sensitivity = 0.70, Specificity = 0.77) (Figure 3A).

External validation of the nomogram model in an independent data set of 501 patients with the seven relevant variables showed strong discrimination. The ROC curve of the nomogram is shown in Figure 3B with an AUC of 0.87 (95% CI = 0.83–0.91, Sensitivity = 0.90, Specificity = 0.75), which was higher than those of the former nomogram (AUC = 0.83, 95% CI = 0.78–0.87, Sensitivity = 0.78, Specificity = 0.76) and GGT alone (AUC = 0.81, 95% CI = 0.76–0.85, Sensitivity = 0.80, Specificity = 0.70). Based on Delong's test for two ROC curves, the former nomogram was not significantly higher than using GGT alone (*P* = 0.23). In comparison, the nomogram was significantly higher compared to the former one (*P* = 0.037) and GGT alone (*P* < 0.0035). The C-index of the newly built model is 0.87, which showed excellent discriminative ability.

The calibration curve showed a close resemblance of the predicted and the actual BA probabilities, manifesting that the nomogram's BA predictability was in favorable accordance with the actual observation, with reference to the probability of BA (Figure 3C). Nevertheless, the DCA curve was carried out to compare the clinical benefits of the novel nomogram with the previous one and GGT alone for BA screening (Figure 3D). A net benefit of DCA showed 0.54 at 80% threshold possibility of the nomogram, which was superior to our previous nomogram (0.49) and GGT alone (0.45).

In summary, the novel nomogram shows better BA early screening performance than the former nomogram and GGT alone.

## Discussion

Early screening of BA is of vital importance due to the disease's tremendous impact on infants' prognosis. Early screening and diagnosis of BA could accelerate choice making between physicians and patients' legal guardians. Thus, more advantageous and convenient methods for early detection are warranted to make an early BA screening. In 2018, we published a nomogram that can facilitate early non-invasive diagnosis of BA, which has been utilized in several other studies in the past 2 years. However, the former nomogram only takes liver function characteristics and patients' basic information to develop the screening model. Hence, we conducted this research to update the nomogram for early screening of BA. The novelties of our new nomogram for early screening of BA using a laboratory marker and US features are as follows:

(1) The newly constructed early BA screening nomogram model has been developed and validated based on 1,502 patients' data collected by the Children's Hospital of Fudan University, National Children's Medical Center. This is the largest BA patient cohort that could represent the majority of BA patients. Therefore, our nomogram may become a readily used BA screening tool for a hospital in China.
(2) The AUC of this newly built nomogram for predicting BA is 0.87, which ranks among the best in the published studies for the record (Figure 3B). The current nomogram had higher sensitivity if the same specificity was required. The patient's gender, serum levels of ALP, DBIL, GGT, and US features like no and poor fasting gallbladder visibility and no and poor fasting gallbladder filling were remarkably higher in BA patients. In contrast, the weight was not relatively significant to compare to the former results we published. This nomogram is the first screening tool that combines laboratory markers and US features together, providing a more accurate and convenient method for clinicians to use. Besides, all of the regular tests are easily obtained from any hospital regardless of geographic location restriction.
(3) The nomogram we proposed is the latest prediction tool with detailed and prudent explication in the stage of model development, validation, and performance assessment. Consequently, our intuitive and transparent model could be tested, validated, and applied in different clinical conditions regardless of the geographic locations and boundaries. Furthermore, this nomogram provides an additional method to evaluate and compare with previously constructed prediction approaches and possible newly developed models in the near future.

BA, characterized by acute and chronic inflammation within the extrahepatic and intrahepatic bile ducts resulting in progressive liver cirrhosis, requires a credible and accurate early screening and diagnosis for prompt surgical drainage (1). Commonly, the early confirmation of BA diagnosis is still adjudicated by surgical cholangiography and intraoperative liver biopsy (15). Besides, precise early BA screening and diagnosis could avoid unnecessary examinations and adverse procedures, which would prolong patients' recovery time and required intensive post-operation care (6). Even though several diagnostic markers and prediction models have been proposed to facilitate effective BA diagnosis, all of them have displayed either unsatisfactory sensitivity and specificity or limited applicability or accessibility in the hospital setting (16–19). The novel nomogram established from our study has been demonstrated as more practicable and precise for BA screening compared with previously proposed prediction models and has potential for clinical application in the future.

In 1996, Choi first reported using the triangular cord sign to distinguish BA from other causes of neonatal cholestasis, of which high diagnostic accuracy of 98% was achieved if the typical triangular cord sign was precisely analyzed (20). A systematic review from Yoon et al. reported that abnormal GB morphologic characteristics, non-visualization of the common bile duct, and the presence of hepatic subcapsular flow showed satisfactory diagnostic accuracy in the diagnosis of BA (21). However, Lee et al. observed a sensitivity of 14% in BA diagnosis when infant patients were younger than 80 days (22). Since the report of triangular cord sign, many other more studies started using sonographic features like hepatic artery enlargement to assist BA early diagnosis. Prior studies have noted that the sensitivity and specificity of the enlargement of hepatic artery varied from 72 to 100% and from 70 to 89%, respectively (6, 23). The main limitations of the triangular cord sign and hepatic artery enlargement are the requirement for qualified and experienced pediatric radiologists and strictly following the detailed US protocol, which is hard to perform in the real world. Previous studies evaluated that non-identifiable GB, small length GB, abnormal shape and morphology, and non-contraction of BA are sensitive and specific US features in BA early diagnosis. Additionally, in our study, we identified that FGBV and FGBF were statistically significant in the early diagnosis of BA (*P* < 0.01). We believe that GB and FGBF are more accessible to detect than the triangular cord signs or the hepatic enlargement during the early phase assessment, which is essential to boost the early BA detection before invasive procedures are performed.

Other imaging techniques such as shear-wave elastography (SWE), MRI, and diffusion tensor imaging (DTI) were reported to be helpful in BA early diagnosis. A study reported by Abdel Razek et al. investigated the DTI parameters of the hepatic parenchyma to distinguish BA and Alagille syndrome (ALGS). The DTI parameters such as fractional anisotropy (FA) and mean diffusivity (MD) of the liver showed higher diagnostic performance in BA early diagnosis, which indicates that DTI could be the extra non-invasive imaging tool for BA from ALGS (24). However, the cost of the ultrasound examination is much lower than that of MRI or DTI. Therefore, screening and diagnostic models based on ultrasonography and laboratory tests may have further clinical application, especially in developing countries.

According to our study, all clinical characteristics and routine tests were documented and screened since the patients were admitted to our center before the surgical procedure. In general, all parameters were easily obtained and contributed to constructing a non-invasive early screening model. Moreover, these parameters were easily obtained and could be evaluated in various hospital settings regardless of sophisticated equipment. The majority of the 14 parameters reported in our research were nutritional status, liver laboratory function, and sonographic features, which showed consistency with the results of previous articles (25, 26). BA patients are usually accompanied by poor liver function and different stages of liver fibrosis. The results of malnutrition status, weakened immunity, coagulative dysfunction, and decreased hemoglobin level might be explained by liver damages and cirrhosis. According to these results, we can infer that total bilirubin, direct bilirubin, and GGT were the most statistically essential parameters. Thus, more BA severity with momentous bile duct obstruction, liver damage, and dysfunction can be suggested.

Furthermore, clinicians could perform an individualized screening for predicting the probability of BA with the convenient early BA screening nomogram. After the calculation by the nomogram and if the patient has a high probability of potential biliary atresia, the clinicians should suggest that the patient receive IOC as soon as possible to confirm the diagnosis and reduce the awaiting time of the Kasai procedure. Moreover, there is no need for urgent exploration for the patients with lower probability, and more investigations should be done to identify the diagnosis.

The primary limitation of this study was rooted in the retrospective and single-center nature. Hence, a prospective study of a larger scale and a multicenter investigation is called to validate our novel nomogram further. An additional uncontrolled factor is GB abnormality, a subjective assessment variable, of which GB abnormality identification primarily relied on the operator's experience and subjective judgment. Therefore, a further objective and systematic approach to documenting GB abnormalities is required to improve US features' diagnostic value.

To conclude, our novel nomogram offers an initial screening tool for BA with better screening performance than current approaches despite its exploratory nature. Our study provides a more convenient and accurate screening method, specifically in a rural and less developed area with limited expertise. Thus, we recommend its practice in all neonatal obstructive jaundice cases in whom BA is suspected.

## Data Availability Statement

The raw data supporting the conclusions of this article will be made available by the authors, without undue reservation.

## Ethics Statement

The studies involving human participants were reviewed and approved by Ethics Committee of the Children's Hospital of Fudan University. Written informed consent to participate in this study was provided by the participants' legal guardian/next of kin. Written informed consent was obtained from the minor(s)' legal guardian/next of kin for the publication of any potentially identifiable images or data included in this article.

## Author Contributions

SZ and RD made the design of the study. Material preparation, data collection, and statistical analysis were performed by SD, YS, YW, GC, SS, and RD. The first version of the manuscript was written by SD, YS, and YW. The critical revision was made by SS, GC, SZ, and RD. All authors have read and approved the ultimate version of the manuscript, conducted the initial conception, design of the study, and revised their comments on previous editions of the manuscript.

## Conflict of Interest

The authors declare that the research was conducted in the absence of any commercial or financial relationships that could be construed as a potential conflict of interest.

## Abbreviations

ALB: Albumin
ALGS: Alagille Syndrome
ALP: Alkaline phosphatase
ALT: Alanine aminotransferase
AST: Aspartate transaminase
AUC: Area under the ROC
BA: Biliary atresia
CBD: Common Bile Duct
CBDV: Common Bile Duct Visibility
CI: Confidential interval
DBIL: Direct bilirubin
DTI: Diffusion Tensor Imaging
FA: Fractional Anisotropy
FGBF: Fasting gallbladder filling
FGBV: Fasting gallbladder visibility
GB: Gallbladder
GGT: Gamma-glutamyl Transpeptidase
GLB: Globulin
IOC: intraoperative cholangiography
MD: Mean Diffusivity
NOJ: Neonatal Obstructive Jaundice
OR: Odds ratio
ROI: Region-of-interest
ROC: Receiver operating characteristic
SWE: Shear-wave elastography
TBA: Total bile acid
TCS: Triangular Cord Sign
US: Ultrasonography.
