# Supplementary material for: Development and Assessment of Screening Nomogram for Biliary Atresia Based on Hepatobiliary Ultrasonographic Features
Source: Front Pediatr. 2021 May 17;9:625451. doi: 10.3389/fped.2021.625451 (PMC8165198; doi:10.3389/fped.2021.625451)
Supplement: Supplementary file 1 [file Table_1.docx]

| **Supplemental Table1. Base Line Clinicopathologic Characteristics of the Training Data Set and Validation Data Set** | | | | | | | | | | | | | |
| --- | --- | --- | --- | --- | --- | --- | --- | --- | --- | --- | --- | --- | --- |
|  |  | Characteristics of Training Data Set (N=1001) | | | |  |  | Characteristics of Validation Data Set (N=501) | | | | | |
|  |  | non-BA | | BA | | Test | P-value | non-BA | | BA | | Test | P-value |
| Gender | female | 42 | 26.8% | 411 | 48.7% | Chi-squared | <0.01 | 26 | 29.5% | 221 | 53.5% | Chi-squared | <0.01 |
|  | male | 115 | 73.2% | 433 | 51.3% |  |  | 62 | 70.4% | 192 | 46.4% |  |  |
|  |  |  |  |  |  |  |  |  |  |  |  |  |  |
| Size of liver | Normal | 59 | 37.6% | 234 | 27.7% | Chi-squared | 0.01 | 31 | 35.2% | 117 | 28.3% | Chi-squared | <0.01 |
|  | Enlargement | 98 | 62.4% | 610 | 72.3% |  |  | 57 | 64.7% | 296 | 71.7% |  |  |
|  |  |  |  |  |  |  |  |  |  |  |  |  |  |
| Fasting gallbladder visibility | No | 11 | 7.0% | 242 | 28.7% | Chi-squared | <0.01 | 9 | 10.2% | 113 | 27.4% | Chi-squared | <0.01 |
|  | Yes | 146 | 93.0% | 602 | 71.3% |  |  | 79 | 89.7% | 300 | 72.6% |  |  |
|  |  |  |  |  |  |  |  |  |  |  |  |  |  |
| Fasting Gallbladder filling | No | 45 | 28.7% | 398 | 47.2% | Chi-squared | <0.01 | 29 | 33.0% | 198 | 47.9% | Chi-squared | <0.01 |
|  | Poor | 34 | 21.7% | 234 | 27.7% |  |  | 14 | 15.9% | 114 | 27.6% |  |  |
|  | Relatively Poor | 43 | 27.4% | 169 | 20.0% |  |  | 24 | 27.3% | 80 | 19.4% |  |  |
|  | Yes | 35 | 22.3% | 43 | 5.1% |  |  | 21 | 23.9% | 21 | 5.1% |  |  |
|  |  |  |  |  |  |  |  |  |  |  |  |  |  |
| Common bile duct visibility | Invisible | 40 | 25.5% | 307 | 36.4% | Chi-squared | <0.01 | 24 | 27.2% | 145 | 35.1% | Chi-squared | <0.01 |
|  | Unclear | 3 | 1.9% | 73 | 8.6% |  |  | 2 | 2.0% | 39 | 9.4% |  |  |
|  | Fine | 82 | 52.2% | 397 | 47.0% |  |  | 41 | 46.5% | 192 | 46.5% |  |  |
|  | Normal | 32 | 20.4% | 67 | 7.9% |  |  | 21 | 23.9% | 37 | 9.0% |  |  |
|  |  |  |  |  |  |  |  |  |  |  |  |  |  |
| Age |  | 73.1±32.2 | 53.0 (67.0, 83.0) | 70.8±30.6 | 53.0 (65.0, 79.0) | Wilcoxon test | 0.21 | 73.4±27.7 | 65.5 (56.0, 84.8) | 74.5±39.9 | 64.0 (52.0, 82.0) | Wilcoxon test | 0.27 |
|  |  |  |  |  |  |  |  |  |  |  |  |  |  |
| Weight* |  | 4.9±1.9 | 4.7 (4.0, 5.5) | 4.8±1.2 | 5.0 (4.0, 5.5) | Wilcoxon test | <0.01 | 4.74±2.2 | 5.0 (4.0，5.0) | 4.8±1.0 | 5.0 (4.0, 5.5) | Wilcoxon test | 0.06 |
|  |  |  |  |  |  |  |  |  |  |  |  |  |  |
| ALB* |  | 40.4±24.2 | 38.8 (36.4, 41.2) | 51.4±97.6 | 38.9 (36.7, 41.3) | Wilcoxon test | 0.57 | 38.4±4.2 | 38.6 (35.4, 40.6) | 52.0±95.6 | 39.2 (37.0, 41.4) | Wilcoxon test | 0.09 |
|  |  |  |  |  |  |  |  |  |  |  |  |  |  |
| ALP* |  | 694.2±357.3 | 607.0 (464.0, 842.0) | 589.2±239.5 | 565.5 (446.0, 698.0) | Wilcoxon test | <0.01 | 650.4±277.6 | 591.5 (461.8, 807.5) | 594.9±236.0 | 573.0 (439.0, 732.0) | Wilcoxon test | 0.19 |
|  |  |  |  |  |  |  |  |  |  |  |  |  |  |
| AST* |  | 178.6±129.0 | 141.0 (95.0, 229.0) | 170.9±122.1 | 146.0 (96.8, 206.2) | Wilcoxon test | 0.73 | 193.7±157.8 | 145.5 (102.0, 255.5) | 177.0±142.0 | 138.0 (97.0, 217.0) | Wilcoxon test | 0.50 |
|  |  |  |  |  |  |  |  |  |  |  |  |  |  |
| DBIL* |  | 103.7±37.7 | 100.3 (78.3, 125.7) | 114.3±51.1 | 109.5 (88.4, 125.7) | Wilcoxon test | <0.01 | 108.0±46.9 | 96.0 (75.0, 135.2) | 109.6±34.1 | 103.9 (87.6, 125.9) | Wilcoxon test | 0.14 |
|  |  |  |  |  |  |  |  |  |  |  |  |  |  |
| GGT* |  | 316.9±383.7 | 181.0 (101.0, 352.0) | 760.6±601.1 | 581.5 (296.0, 1092.0) | Wilcoxon test | <0.01 | 251.1±220.1 | 191.0 (112.0, 300.2) | 700.5±541.3 | 538.0 (269.0, 980.0) | Wilcoxon test | <0.01 |
|  |  |  |  |  |  |  |  |  |  |  |  |  |  |
| GLB* |  | 15.7±4.8 | 14.9 (12.5, 17.5) | 15.7±4.1 | 15.0 (13.0, 17.6) | Wilcoxon test | 0.40 | 15.2±3.8 | 14.9 (12.4, 17.0) | 16.0±3.8 | 15.4 (13.4, 18.0) | Wilcoxon test | 0.07 |
|  |  |  |  |  |  |  |  |  |  |  |  |  |  |
| TBA* |  | 139.6±64.5 | 131.3 (99.9, 171.4) | 137.9±59.5 | 128.4 (99.9, 164.1) | Wilcoxon test | 0.73 | 139.5±136.3 | 119.6 (90.5, 150.5) | 138.6±84.3 | 124.8 (98.9, 157.8) | Wilcoxon test | 0.18 |
|  |  |  |  |  |  |  |  |  |  |  |  |  |  |
| TBIL* |  | 157.3±55.6 | 151.7 (118.0, 190.5) | 164.8±49.5 | 157.6 (131.5, 187.2) | Wilcoxon test | 0.08 | 161.7±69.7 | 141.4 (114.7, 201.5) | 162.3±46.9 | 155.4 (132.3, 185.5) | Wilcoxon test | 0.12 |

* Data are presented as mean ± SD and median (Q1, Q3). Other data are present as frequency with percentage
