# Supplementary figures and images for: Development and Assessment of Screening Nomogram for Biliary Atresia Based on Hepatobiliary Ultrasonographic Features
Source: Front Pediatr. 2021 May 17;9:625451. doi: 10.3389/fped.2021.625451 (PMC8165198; doi:10.3389/fped.2021.625451)

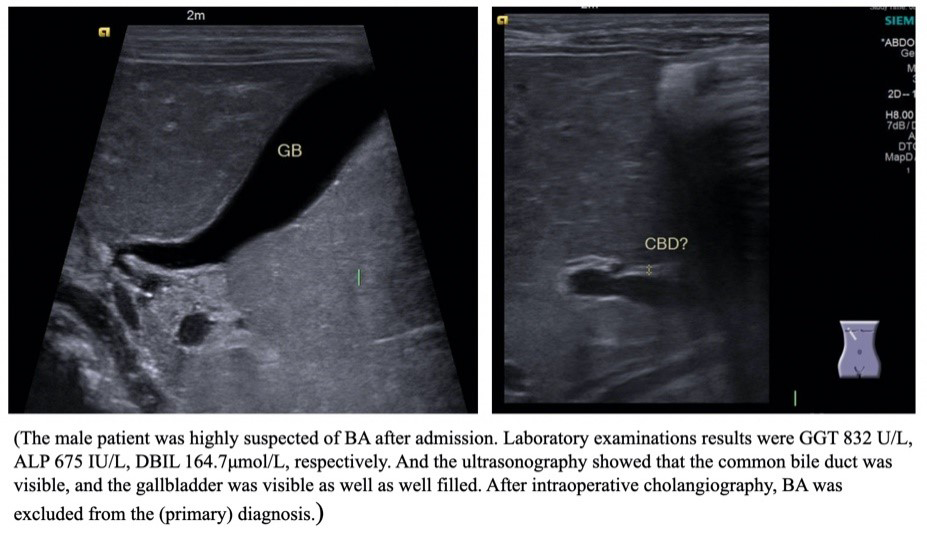

Supplement: Supplementary file 2 [file Image_1.TIF]
